# Neutrophil extracellular traps in patients with pulmonary tuberculosis

**DOI:** 10.1186/s12931-017-0663-1

**Published:** 2017-10-30

**Authors:** Anne Jan van der Meer, Sacha Zeerleder, Dana C. Blok, Liesbeth M. Kager, Ivar O. Lede, Wahid Rahman, Rumana Afroz, Aniruddha Ghose, Caroline E. Visser, Abu Shahed Md Zahed, Md Anwar Husain, Khan Mashrequl Alam, Pravat Chandra Barua, Mahtabuddin Hassan, Md Abu Tayab, Arjen M. Dondorp, Tom van der Poll

**Affiliations:** 1 Centre for Experimental and Molecular Medicine, Academic Medical Center, University of Amsterdam, Meibergdreef 9, Room G2-130, 1105 AZ Amsterdam, The Netherlands; 2 Department of Haematology, Academic Medical Center, University of Amsterdam, Amsterdam, The Netherlands; 3 Department of Microbiology, Academic Medical Center, University of Amsterdam, Amsterdam, The Netherlands; 4 Division of Infectious Diseases, Sanquin Blood Supply and Landsteiner Laboratory of the Academic Medical Center, University of Amsterdam, Amsterdam, The Netherlands; 5 Department of Immunopathology, Chittagong Medical College & Hospital (CMCH), Chittagong, Bangladesh; 6 Department of Internal Medicine, Chittagong Medical College & Hospital (CMCH), Chittagong, Bangladesh; 7 Department of Microbiology, Chittagong Medical College & Hospital (CMCH), Chittagong, Bangladesh; 8 National TB Control & Leprosy Elimination Program, Dhaka, Bangladesh; 9 Chittagong General Hospital, Chittagong, Bangladesh; 10 Mahidol Oxford Tropical Medicine Research Unit, Mahidol University, Bangkok, Thailand

## Abstract

Tuberculosis is a devastating infectious disease causing many deaths worldwide. Recent investigations have implicated neutrophil extracellular traps (NETs) in the host response to tuberculosis. The aim of the current study was to obtain evidence for NETs release in the circulation during human tuberculosis. For this we measured the plasma concentrations of nucleosomes in conjunction with neutrophil elastase, in 64 patients with active pulmonary tuberculosis and 32 healthy controls. Patients with active tuberculosis had elevated plasma levels of nucleosomes and elastase when compared with local healthy blood donors. Furthermore nucleosome and elastase levels showed a positive correlation. These findings provide the first evidence for the release of NETs in the circulation of patients with active pulmonary tuberculosis.

## To the editor,

The global burden of tuberculosis is high, with 10.4 million cases and 1.4 million deaths in 2015. Especially in Bangladesh the disease is highly prevalent, with 225 new cases per 100,000 citizens in 2015 (Global Tuberculosis Report World Health Organization 2016). While CD4+ T cells and type 1 cytokines are well-studied components of protective immunity after infection with the causative agent *Mycobacterium (M.) tuberculosis*, recent investigations also point to a role for neutrophils in the host response during tuberculosis [1]. Neutrophils are the cell types predominantly infected with *M. tuberculosis* in patients with active pulmonary tuberculosis [2] and the blood transcriptional signature associated with human tuberculosis is driven by neutrophil-specific gene expression profiles [3]. While neutrophils are not able to kill *M. tuberculosis* [4], they may assist in host defense and containment of the infection by interacting with other cell types [1]. Neutrophils can release neutrophil extracellular traps (NETs) composed of nucleosomes, histones and granular proteases such as elastase [5]. NETs can exert antimicrobial effects, in part mediated through their ability to trap pathogens [5]. Recent investigations have implicated NETs in the host response to tuberculosis. Neutrophils release NETs upon incubation with *M. tuberculosis* in vitro [6–8] and sputum of patients with tuberculosis contains NETs [8]. The aim of the current study was to obtain evidence for NETs release in the circulation during human tuberculosis. For this we measured the plasma concentrations of nucleosomes, a suitable marker for NETs formation in plasma in humans [9, 10], in conjunction with neutrophil elastase, in patients with active pulmonary tuberculosis.

Sixty one patients (age 28 [22–44] years) and 32 healthy blood donors (30 [24–35] years) were recruited in the Tuberculosis Clinic of Chittagong General Hospital and the Chittagong Medical College & Hospital, Chittagong, Bangladesh (Table 1). The study was approved by the National Research Ethics Committee, Bangladesh Medical Research Council, Bangladesh and the Oxford Tropical Research Ethics Committee, University of Oxford, Oxford, UK (OXTREC 35–09). Written informed consent was obtained from all study subjects or next-of-kin by a native Bengali speaker. These subjects were part of a larger population in which the expression of Toll-like receptor regulators was studied [11]. Inclusion and exclusion criteria have been reported in detail [11]. The study subjects were all newly registered patients who had not (yet) received therapy at the time of enrolment. On-site tuberculosis confirmation was defined by a minimum of two out of three positive Ziehl-Neelsen stained sputum samples collected on two consecutive days. *M. tuberculosis* infection was confirmed by polymerase chain reaction (GeneXpert, Cepheid, Solna, Sweden). White blood cells were manually counted by blood smear. All patients and controls were tested for human immunodeficiency virus (Determine® HIV 1/2 test; Almere, Tilburg, The Netherlands). Nucleosomes, elastase-α1-antitrypsin and factor VII–activating protease (FSAP)-α2-antiplasmin complexes were measured in citrate-anticoagulated plasma by enzyme-linked immunosorbent assays as described [10, 12]. The nucleosome ELISA uses a catching antibody that recognizes histone 3 and a detection antibody that recognizes an epitope exposed on complexes of histone 2A, histone 2B and double stranded DNA [10]. Comparisons between groups were performed by Mann-Whitney U tests and correlations were calculated by Spearman’s rho test using GraphPad Prism version 5.01 (GraphPad Software, San Diego, CA). Data are presented as medians with interquartile ranges. *P* < 0.05 was considered statistically significant.

**Table 1:** Patient characteristics

	Healthy controls *n* = 32	Primary TB *n* = 61
Demographics		
Age (years)	30 (24–35)	28 (22–43,5)
Male sex (*n*)	25 (78%)	44 (72%)
Smoker (*n*)	8 (25%)	30 (49%)
Affected family member with TB (*n*)	0 (0%)	6 (10%)
BCG-vaccinated (*n*)	20 (63%)	21 (34%)
HIV-positive (*ii*)	0 (0%)	2 (3%)
Symptoms		
Fever (*n*)	0 (0%)	61 (100%)
Night sweats (*n*)	0 (0%)	23 (38%)
Weight loss (*n*)	0 (0%)	44 (72%)
Fatigue (*n*)	1 (3%)	32 (52%)
Shortness of breath (*n*)	1 (3%)	7 (11%)
Productive cough (*n*)	3 (9%)	56 (92%)
Signs		
Temperature (°C)	36.9 (36.5–37.1)	37.2 (36.9–38.1)***
MAP (mmHg)	83 (79–93)	80 (70–87)*
Heart rate (bpm)	80 (78–84)	90 (80–100)***
Respiratory rate (brpm)	20 (20–24)	24 (22–28)***
BMI (w/1^2^)	24.3 (22.7–26.0)	17.6 (15.6–19.6)***

Abbreviations: *BMI* body mass index, expressed as weight (w) divided by length (l)2; *bpm* beats per minute, *brpm* breaths per minute, *MAP* mean arterial blood pressure, *n* total number, *TB* tuberculosis. Percentages given are within study group. Data are medians with interquartile ranges. *P < 0·05, ***P < 0·001 for the difference between primary TB or recurrent TB-patients versus controls

**Fig. 1:**
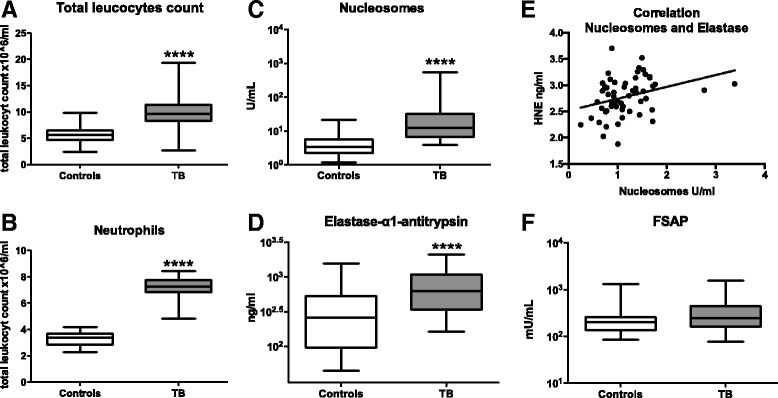
Leukocyte counts, neutrophil counts, plasma levels of nucleosomes, elastase and factor VII–activating protease in patients with pulmonary. Total leukocyte counts (**a**) and neutrophil counts (**b**) in blood; plasma concentrations of nucleosomes (**c**) and elastase-α1-antitrypsin (**d**), the correlation between nucleosome and elastase levels as determined by Spearman’s rho test (**e**); plasma levels of factor VII–activating protease (FSAP)-α2-antiplasmin complexes (**f**). Data are from healthy blood donors (*n* = 32) and tuberculosis patients (*n* = 61), and shown as box-and-whisker diagrams depicting the median, the smallest observation, lower quartile, median, upper quartile and largest observation. ****P < 0.0001

Demographic data, together with clinical signs and symptoms, are shown in the Table 1. Patients did not have significant comorbidities. Relative to healthy controls, tuberculosis patients had leucocytosis (Fig. 1, panel A) caused by a rise in neutrophil counts (Fig. 1, panel B). Patients with active tuberculosis showed elevated plasma levels of nucleosomes when compared with local healthy blood donors (111.7 U/ml versus 5.4 U/ml, *P* < 0.0001; Fig. 1, panel C). Plasma elastase concentrations were also higher in patients with tuberculosis (854.0 ng/ml versus 397.3 ng/ml in healthy donors, P < 0.0001; Fig. 1, panel D), and nucleosome and elastase levels showed a positive correlation (Spearman’s rho 0.37, *P* < 0.005; Fig. 1, panel E). Tuberculosis patients more often smoked than healthy controls (49% versus 25% respectively, Table 1) and a recent study showed nicotine has NETs inducing properties [13]. However, comparing nucleosomes and elastase levels between smokers and non-smokers within the tuberculosis patient group showed no significant differences (data not shown).

Elastase is an important component of NETs that can assist in the degradation of bacterial virulence factors [5]. Upon activation of neutrophils elastase translocates to the nucleus, where it aids in chromatin decondensation leading to NETosis [5]. Additionally, elastase can activate macrophages and increase their capacity to kill intracellular pathogens [14]. Moreover, macrophages can bind *M. tuberculosis* induced NETs and ingest elastase from neutrophils, leading to the release of increased amounts of proinflammatory cytokines [7] like we found in the tuberculosis patients included in this study [11]. Together these data suggest that during tuberculosis elastase could be involved in an interaction between NETotic cells and macrophages resulting in a proinflammatory and protective immune response. Notably, this interaction between NETs, elastase and other host cells may also be associated with collateral damage, as has been described in a variety of infectious and inflammatory diseases [5].

Two patients (3%) and none of the blood donors tested positive for HIV (Table 1). The low prevalence of HIV positive tuberculosis in our cohort is in accordance with data from the World Health Organization derived from Bangladesh (Global Tuberculosis Report World Health Organization 2016 and allowed us to study the association between tuberculosis and NETs formation without the potential bias introduced by concurrent HIV infection. Indeed, HIV, like *M. tuberculosis* (6–8), triggers NETs release from neutrophils [15].

FSAP circulates as a single-chain molecule and is activated upon contact with apoptotic and necrotic cells [16]. Activated FSAP mediates the release of nucleosomes from apoptotic cells [16]. Acute infections, particularly sepsis, are associated with an increased activity of FSAP [12]. In our cohort plasma FSAP activation as measured by FSAP-α2-antiplasmin complexes did not differ between tuberculosis patients and controls (Fig. 1, panel F), suggesting that FSAP may not contribute to nucleosome release in this population. The absence of increased FSAP activity further suggests that nucleosome release during tuberculosis is caused by NETosis rather than by apoptosis or necrosis [17]. In this context it is interesting to note that neutrophil apoptosis is not required for *M. tuberculosis* induced NETosis [7].

Our study has limitations. Duration of symptoms was not documented. Our data do not prove that plasma nucleosomes are solely or predominantly derived from neutrophils. The poor resource setting and the relatively small sample size precluded careful evaluation between disease severity and nucleosome levels. In addition, the lack of follow up samples excluded analyses of sequential nucleosome levels and disease course. None of the patients died from tuberculosis.

In conclusion, we here provide evidence for the release of NETs in the circulation of patients with active pulmonary tuberculosis, corroborating previous studies showing the capacity of *M. tuberculosis* to induce NETs release from neutrophils in vitro [6–8]. Our study does not reveal whether plasma nucleosomes originate from infected lesions or from systemic release, although the concurrently elevated plasma elastase levels and the fact that tuberculosis is associated with altered gene expression in blood neutrophils [3] suggest that the latter option is more likely. The exact role of NETs in the host response during tuberculosis, whether contributing to protective immunity or causing collateral damage, requires further investigation.
